# Carbohydrate-Derived Amphiphilic Macromolecules: A Biophysical Structural Characterization and Analysis of Binding Behaviors to Model Membranes

**DOI:** 10.3390/jfb6020171

**Published:** 2015-04-08

**Authors:** Adriana A. T. Martin, Michael Tomasini, Vladyslav Kholodovych, Li Gu, Sven Daniel Sommerfeld, Kathryn E. Uhrich, N. Sanjeeva Murthy, William J. Welsh, Prabhas V. Moghe

**Affiliations:** 1 Department of Pharmacology, Rutgers University, Piscataway, 675 Hoes Lane, Piscataway, NJ 08854, USA; E-Mails: adrianamartin7@gmail.com (A.A.T.M.); kholodvl@ca.rutgers.edu (V.K.); welshwj@rwjms.rutgers.edu (W.J.W.); 2 Department of Biomedical Engineering, Rutgers University, 599 Taylor Road, Piscataway, NJ 08854, USA; 3 Department of Chemical and Biochemical Engineering, Rutgers University, 599 Taylor Road, Piscataway, NJ 08854, USA; E-Mail: toma0052@gmail.com; 4 New Jersey Center for Biomaterials, 145 Bevier Road, Piscataway, NJ 08854, USA; E-Mails: Sommerfeld@dls.rutgers.edu (S.D.S.); Murthy@dls.rutgers.edu (N.S.M.); 5 Department of Chemistry and Chemical Biology, Rutgers University, 610 Taylor Road, Piscataway, NJ 08854, USA; E-Mails: lig@mit.edu (L.G.); keuhrich@rutgers.edu (K.E.U.); 6 OIRT/High Performance and Research Computing, 185 S. Orange Avenue, Newark, NJ 07103, USA

**Keywords:** amphiphilic macromolecule, membrane lipid bilayers, quartz crystal microbalance with dissipation (QCM-D), molecular dynamics simulations, quantitative structure-activity relationship (QSAR) model

## Abstract

The design and synthesis of enhanced membrane-intercalating biomaterials for drug delivery or vascular membrane targeting is currently challenged by the lack of screening and prediction tools. The present work demonstrates the generation of a Quantitative Structural Activity Relationship model (QSAR) to make *a priori* predictions. Amphiphilic macromolecules (AMs) “stealth lipids” built on aldaric and uronic acids frameworks attached to poly(ethylene glycol) (PEG) polymer tails were developed to form self-assembling micelles. In the present study, a defined set of novel AM structures were investigated in terms of their binding to lipid membrane bilayers using Quartz Crystal Microbalance with Dissipation (QCM-D) experiments coupled with computational coarse-grained molecular dynamics (CG MD) and all-atom MD (AA MD) simulations. The CG MD simulations capture the insertion dynamics of the AM lipophilic backbones into the lipid bilayer with the PEGylated tail directed into bulk water. QCM-D measurements with Voigt viscoelastic model analysis enabled the quantitation of the mass gain and rate of interaction between the AM and the lipid bilayer surface. Thus, this study yielded insights about variations in the functional activity of AM materials with minute compositional or stereochemical differences based on membrane binding, which has translational potential for transplanting these materials *in vivo*. More broadly, it demonstrates an integrated computational-experimental approach, which can offer a promising strategy for the *in silico* design and screening of therapeutic candidate materials.

## 1. Introduction

Membrane lipid bilayers provide a natural environment for the immobilization and entrapment of bioactive small molecules and macromolecules. There have been several biotechnological advances to take advantage of the membrane landscape. This includes what is referred to as stealth lipids, which carry a head group tethered to a polyethylene glycol polymer. The membrane binding replicates amphiphilic molecular behavior found in cellular environs, such as bile salts, cholesterol and peptides. Supported lipid bilayers serve as practical model systems for the characterization and optimization of biomolecules, such as amphiphilic macromolecules (AMs), that interact with and bind to cell membranes [1]. A variety of drug delivery systems make use of AMs as functional modifiers of membrane surfaces [2,3,4]. Hence, the impetus for the present study was to gain a more detailed molecular-level understanding of the AM-membrane interactions.

**Figure 1:**
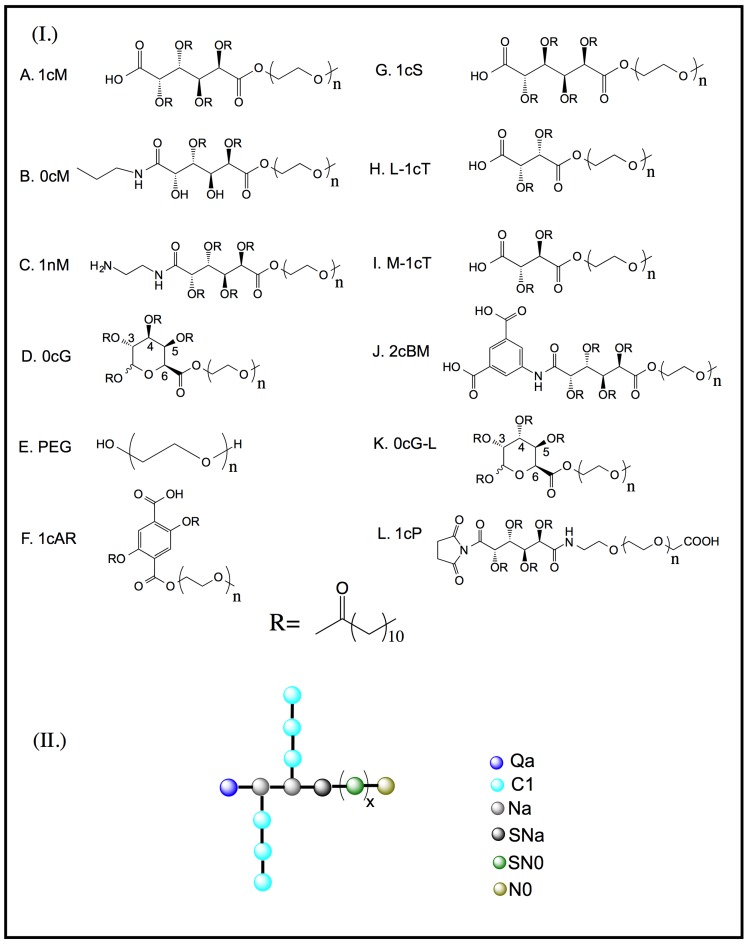
(**I**) Chemical structure of carbohydrate-based backbone versions of amphiphilic macromolecules (AMs), variations include the polarity of the head group (–COOH, NH_2_), number of aliphatic side chains, and aromaticity; (**II**) Simulation beads composed of heavy atoms represented using CG MARTINI force field developed by Marrink *et al*. [9]. The bead types are as follows: Q—charged; P—polar; N—non-polar; C—apolar; *n* or *x* refers to 113 units of poly(ethylene glycol) and truncated PEG length of 45 used to reduce computational cost for the molecular dynamics studies.

The recent development of experimental methods to measure relevant features of permeation and partition into lipid bilayers has advanced the ability of scientists to design and fine tune biological macromolecules. The present study describes the evaluation of a focused set of AMs (Figure 1) in terms of their interaction with and binding to a model lipid membrane bilayer [5,6,7]. These AMs were originally developed as delivery systems, with the designed feature of spontaneous micelle formation in aqueous solution [6]. More recently, studies from our laboratories have established AMs can bind to scavenger receptors and thus block oxidized low-density lipoprotein interaction, thereby mitigating downstream consequences of initial cellular insult [5,8].

**Figure 2:**
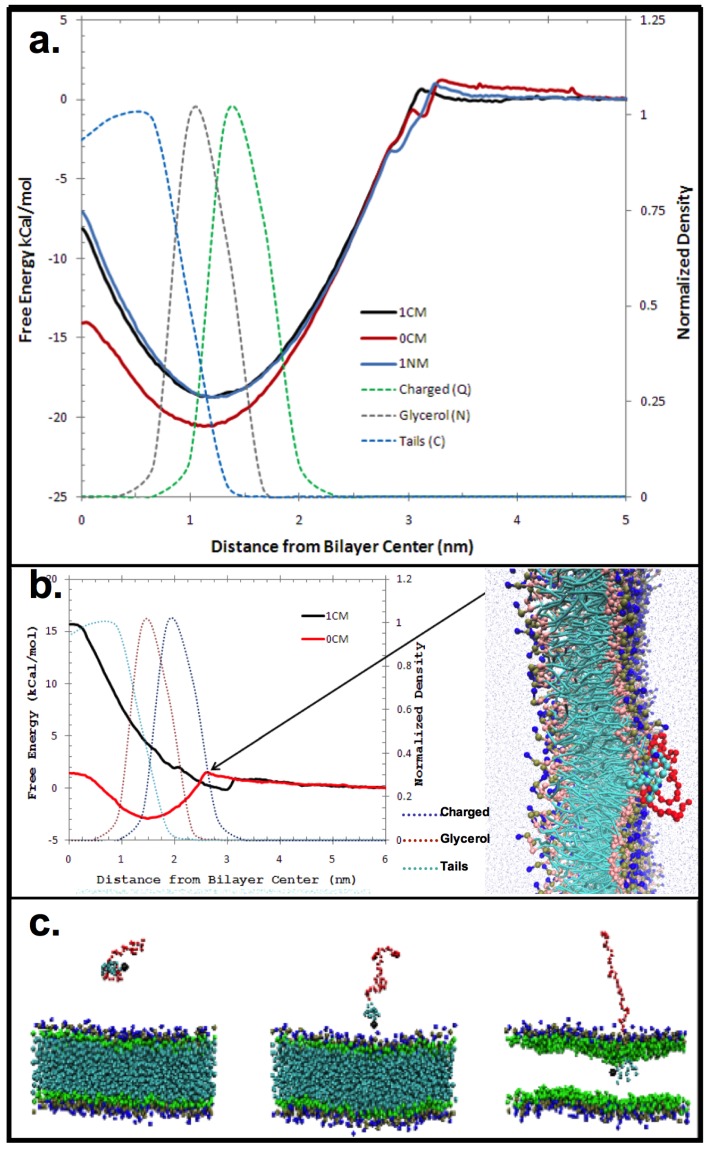
(**a**) Free Energy of insertion for each selected macromolecule into the center of the bilayer. In each case, the Center of Mass (COM) of the non-PEG portion of the polymer was pulled into the center of the bilayer; (**b**) Potential of Mean Force for 1cM and 0cM; (**c**) Relationship between lipophilicity and membrane insertion. CG MD results: insertion of AM into zwitterionic model membrane. The system snapshots show the progression of 1cM insertion into the membrane. The PEG tail of the AM is depicted in red and the variable hydrophobic element or head region is colored blue. Bilayer membrane molecules are shown as green and dark blue.

**Figure 3:**
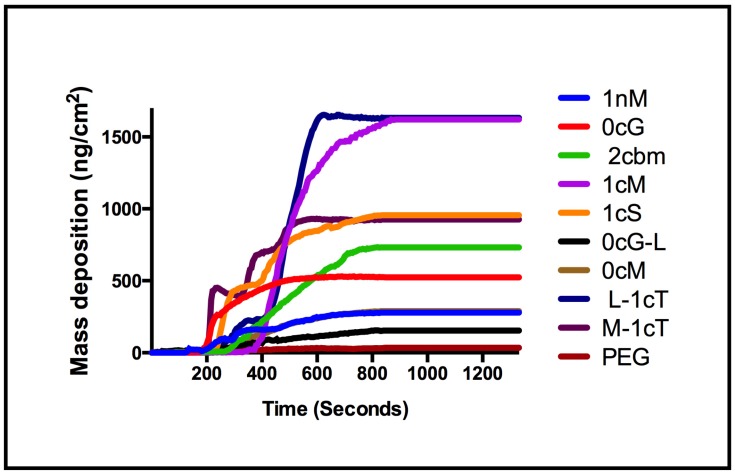
Comparison of mass deposition on a supported lipid membrane for AMs at concentration of 1 × 10^−6^ M.

**Figure 4:**
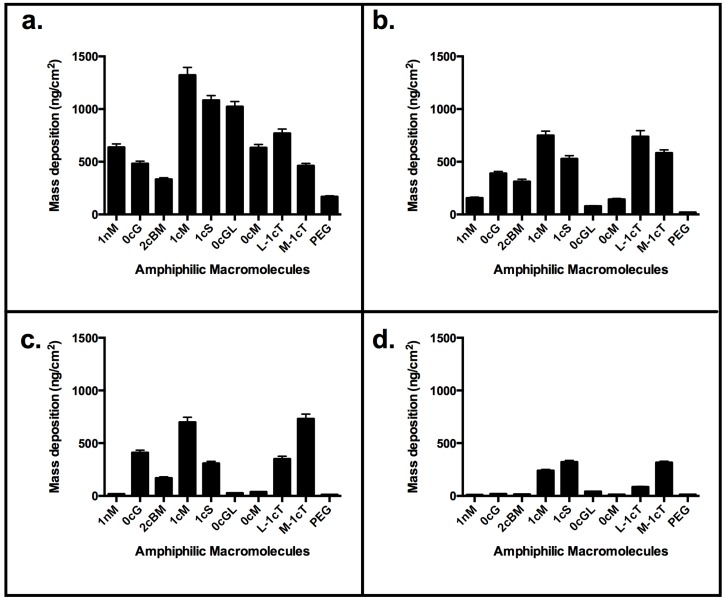
QCM-D results: Comparison of binding to supported lipid bilayer for concentration of AM above and below CMC values of 1 × 10^−7^ M. (**a**) 1 × 10^−5^ M; (**b**) 1 × 10^−6^ M; (**c**) 1 × 10^−7^ M; (**d**) 1 × 10^−8^ M. Stereochemistry influences the overall AM conformation. Best binders include 1cM, 1cS and the analogs of truncated backbone, L-1cT and M-1cT.

**Figure 5:**
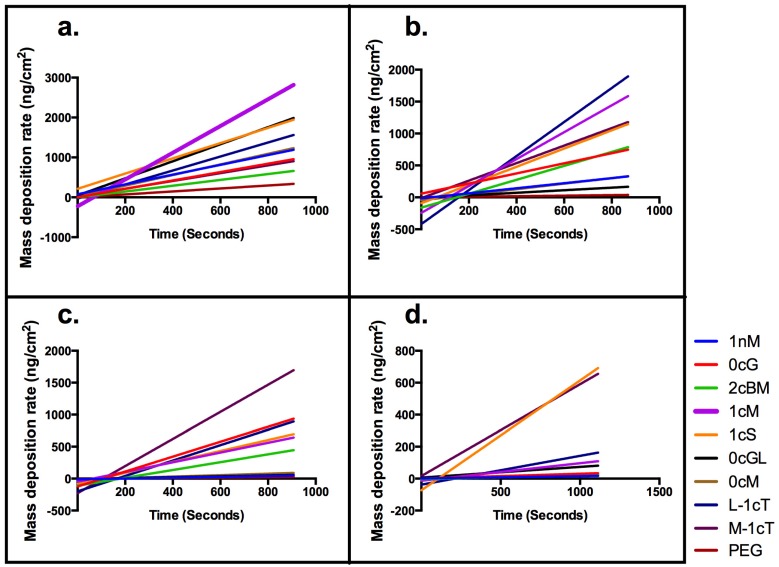
QCM-D mass deposition rate trends. Linear regression lines are plotted.

**Figure 6:**
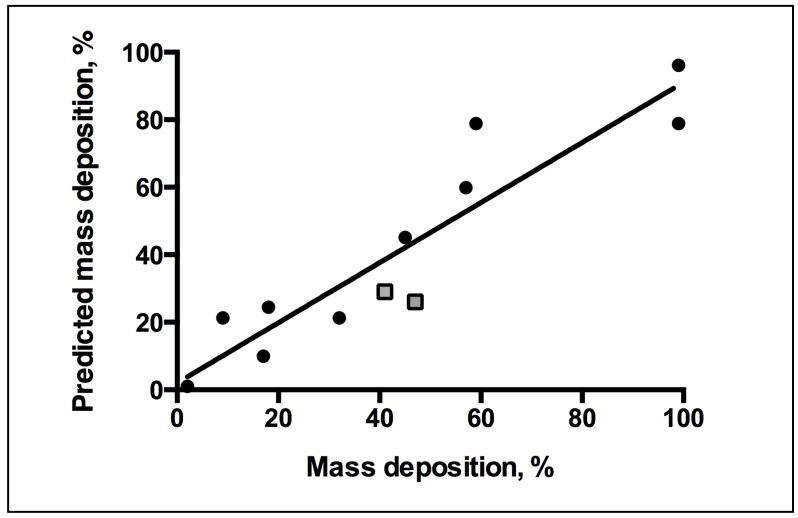
Correlation between predicted *versus* experimental membrane binding outcomes for QSAR linear fit of *R*^2^ = 0.89. QSAR equation related to mass binding and descriptors of macromolecules for statistical analysis of model fit. Key: Square, new test set of mass deposition; Circle, training set mass deposition.

**Figure 7:**
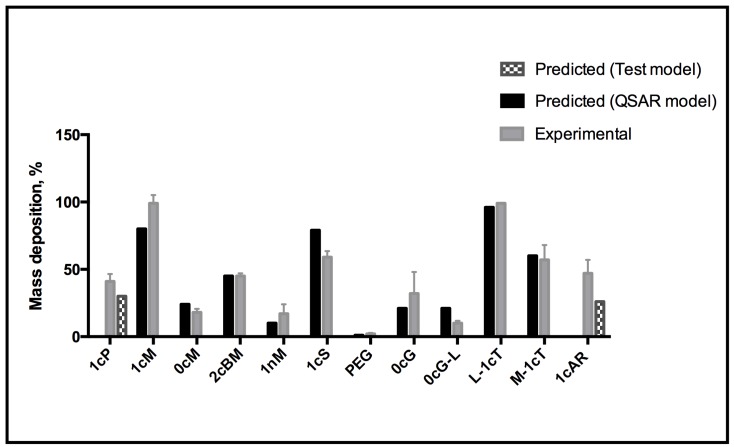
QSAR model for predicting AM membrane association.

A particularly attractive feature of AMs (Figure 1) as drug delivery and surface modifying agents is their ability to interact with lipid membranes. In the present context, computational molecular simulations of multicomponent ensembles entail methods in which AMs are judiciously placed above model lipid bilayer membranes (Figure 2). This provides a powerful tool for probing the fundamental intermolecular interactions between the AMs and lipid bilayers. Complications of this task include the necessity of high resolution and anisotropy of the lipid membrane composition. Toward that end, the present study employed an integrated computational-experimental approach to investigate a set of AMs with respect to their interaction with, and binding to, a model lipid bilayer system (Figure 3, Figure 4 and Figure 5). The primary aim was to elucidate key physicochemical features of these AMs that affect their association with biological lipid membranes [8]. The development of a Quantitative SAR (QSAR) model (Figure 6 and Figure 7) has demonstrated satisfactory findings. The present investigation will ultimately guide the rational design of AMs with optimal membrane binding capabilities for therapeutic applications [10,11].

To gain insight on the influence of AM physicochemical properties on membrane binding, a combination of experimental Quartz Crystal Microbalance with Dissipation (QCM-D) [12] and computational molecular dynamics (MD) simulations methods were employed. QCM-D provides a direct biophysical method for assessing the structural effects on AM binding under physiological conditions. Coarse-grained MD (CG MD) simulations are highly complementary to such experimental methods, as they provide a means to explore the interactions between individual molecules [13]. A defined set of AM structures was investigated, including stereoisomer pairs and close structural analogs, which differ by a single feature (e.g., charge, stereochemistry) [14,15].

QCM-D experiments and CG MD simulations were performed on each AM, thus providing the input data to build a preliminary QSAR model. This method identified key physicochemical features (known as molecular descriptors) of the AMs in relation to percent mass deposition on model lipid membranes. The resultant QSAR model can be useful for guiding the design and optimization of new AMs and for ascertaining key physicochemical features of the AMs that may enhance membrane binding. The descriptors were extracted from each of the AM structures in low-energy conformations. This was generated by CG MD simulations using a scheme known as “reverse mapping”, as described previously [16]. The conversion of coarse-grained structures into atomistic models for subsequent all-atom MD (AA MD) simulations was followed by descriptor generation. This progression of steps efficiently exploited the ability of CG MD simulations to model complex macromolecule-membrane systems under biologically relevant conditions for extended time scales. It also provides the molecular-level resolution of AA MD simulations required for descriptor generation and detailed analysis of AM-membrane interactions [17,18,19,20].

## 2. Results and Discussion

**Figure 8:**
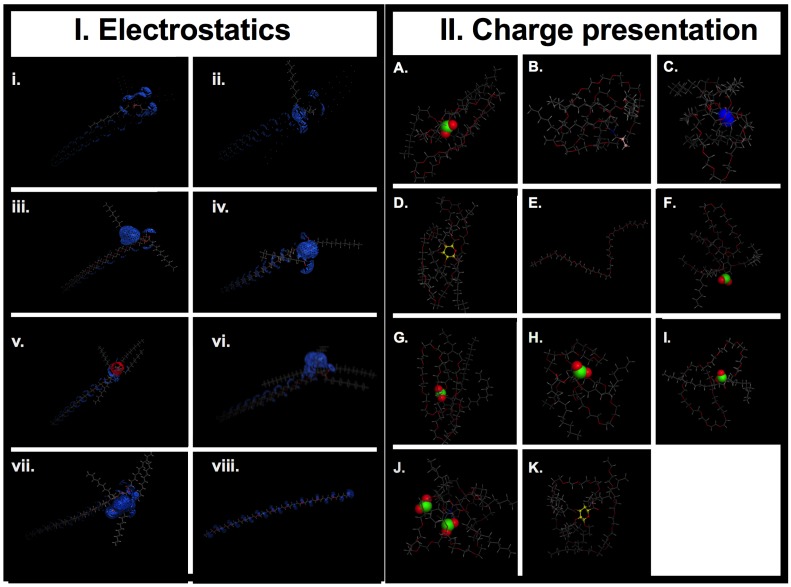
(**I**) Electrostatic surface map for AMs. Generated using MOE (Molecular Operating Environment, 2013.08, Montreal, Canada). Panels: (i) 0cG, (ii) 0cM, (iii) 1cAR, (iv) 1cT, (v) 1nM, (vi) 1cM, (vii) 2cBM, (viii) PEG. Key: Red, Hydrogen bond Acceptor; Blue, Hydrogen bond Donor. (**II**) Charge presentation. Final structural arrangement effect on charge presentation: (A) 1cM has the charge center exposed to solution *versus* (G) 1cS stereoisomer of 1cM has a protected charge group sequestered in a cage like conformation. (A) 1cM, (B) 0cM, (C) 1nM, (D) 0cG, (E) PEG control, (F) 1cAR, (G) 1cS, (H) L-1cT, (I) M-1cT, (J) 2cBM, (K) 0cG-L. Conformations correlate to the structural arrangement of the lowest energy achieved for the run. The green: negative charge; blue: positive charge; pink: neutral carbon capped chain; and yellow: cyclic group.

Molecular descriptors were generated to encode various physicochemical properties of the AM including spatial organization, chemical composition and stereochemistry. Following filtering and prioritization of descriptors based on their information content, QSAR models were constructed using Partial Least Squares (PLS) regression to predict the AM bioactivity (*i.e.*, rate and extent of mass deposition). A statistically strong correlation (*r*^2^ = 0.9, $r_{cv}^{2}$ = 0.7) between predicted and observed values of mass deposition was achieved with four PLS terms (latent variables). Values of the mass deposition were predicted for two additional AMs with varying hydrophobic moieties novel to the QSAR model. The predictions were consistent with experimental QCM-D membrane binding in terms of binding rank order. The low-energy conformations reflected the effects of structural changes on conformation and solvent exposure of charge density (Figure 1 and Figure 8).

The CG MD simulations provided a detailed view of the membrane binding profile of the AM species at the membrane interface (Figure 2). The AMs were found to associate with the lipid bilayer within 10 ns from start of the post-equilibration simulations. The association with the zwitterionic model lipid bilayer was observed to be via insertion of the hydrophobic backbone, with the PEGylated tail projecting outward into bulk water above the membrane (Figure 2). Membrane binding was strongly influenced by the unique structural features (*viz*., charge, stereochemistry) of the AMs. Specifically, the orientation of the aliphatic arms attached to the carbohydrate backbone affect membrane binding. As the thickness of a dipalmitoylphosphatidylcholine (DPPC) bilayer is approximately 4 nm, AM head group to bilayer Center of Mass (COM) distances <2 nm occurring during a simulation were taken to be an insertion event. Our findings show all of the AMs exhibit membrane binding.

A more detailed analysis of membrane penetration was conducted on three AMs; *viz*., 1cM, 1nM and 0cM. For each, the Free Energy of insertion was calculated from the MD simulations (Figure 2a). In each case, the COM of the carbohydrate-based backbone of the AM was placed in the center of the bilayer. Umbrella sampling was used to determine the Free Energy along the reaction coordinate perpendicular to the bilayer. Both 1cM and 1nM adopted an equilibrium position at the junction between the charged choline moiety and the glycerol portion of the bilayer. 0cM, lacking a charged head group, adopted an equilibrium position coincident with the peak in the bilayer of the glycerol group (Figure 2a). This shows the importance of charge for binding to a zwitterionic membrane. The phosphatidylcholine head group of the membrane lipid has a dipole moment, and binding of ions have been suggested to induce conformational changes allowing for ion-dipole interactions with little entropic cost [21,22,23]. As seen in this case, specific interactions can occur at the membrane interface with electrostatic energies dependent on ion polarizability and ionic interactions [24].

The Potential of Mean Force (PMF) for 1cM and 0cM was measured by MD to better understand changes in the Free Energy of the head group and the importance of charge along the reaction coordinate. PMFs are derived from statistical analysis of experimentally observed distributions and frequencies of specific atom-pair interactions. The results showed free energy decreases for 1cM as it nears the zwitterionic outer region of the bilayer but increases slightly for 0cM until it penetrates into the bilayer (Figure 2b). The coarse-grained structures for 1cM and 0cM offer a general assessment of physicochemical trends on binding. The interaction comparison in this instance for charged 1cM *versus* neutral 0cM can be used to illustrate the significance of charge on binding of these macromolecules. The charged group has greater binding than the neutral version otherwise identical.

The AMs (Figure 1, A-L) were studied at the Critical Micelle Concentrations (CMC) for profiles of their membrane binding [25]. The micelles provide a reservoir for AM unimers, whose concentration will depend on the value of the CMC [26]. The shielding effect of the aliphatic arms (Figure 1) on exposure of charge can be clearly demonstrated by the difference in binding between 1cM and 1cS (Figure 4c) at CMC of 10^−7^ M [27]. Visual inspection of the low-energy MD conformations (Figure 8) revealed 1cS adopts a cage-like architecture with the aliphatic chains surrounding the charge point. This phenomenon would explain the reduced binding affinity of 1cS compared with 1cM for which the charged group is exposed [Figure 8(A *versus* G)].

Analogous to 1cS and 1cM, the stereoisomeric pair of M-1cT and L-1cT showed a change of orientation in 3D space, directly affecting membrane binding. Interestingly, L-1cT is similar to 1cM with respect to its terminal charge configuration and stereochemistry of the flanking aliphatic chains (Figure 8). However, the plateau in mass deposition is significantly greater for 1cM and L-1cT (750 and 740 ng/cm^2^) than for their corresponding stereoisomeric pairs 1cS and M-1cT (530 and 580 ng/cm^2^). This pattern can be observed at a concentration of 1 × 10^−6^ M in Figure 3, which compares AM membrane binding over the course of a 15-minute period. Specific stereochemical conformations and charge presentations, as exemplified by 1cM and L-1cT, exhibit significantly enhanced binding association. PEG, which served as a control, exhibited minimal membrane binding according to both the QCM-D experiments and computational studies.

The observed membrane binding of uncharged 0cM is much lower compared with its charged analog 1cM (Figure 4). This result is consistent with our observation CG MD simulation results showing the importance of charge for binding (Figure 2) [28]. It is likely the charge on the AM head group will drive an initial electrostatic interaction with membrane lipids needed for further binding of AMs to the membrane [29,30]. However, due to the higher thermodynamic requirements, an uncharged AM would retain in aqueous solution *versus* binding to the bilayer membrane.

The degree of electrostatic charge distribution is illustrated in Figure 8I. The binding interactions show the density of charge and aliphatic arm conformation relative to the charge locus position in the conformational space. For the AM 0cG and its stereoisomer 0cG-L, which feature a R-substituted aliphatic heterocyclic group in place of the aliphatic side chain of 1cM and related AMs, exhibited lower membrane binding affinity. However, their binding affinity was still appreciable at higher concentrations (Figure 4). The dissimilarity in structures between 0cG or 0cG-L and 1cM preclude a direct structural comparison. However, the binding *versus* concentration trends for these two AMs suggest that these AMs may interact with the membrane bilayer as micelles at higher concentrations. Likewise 2cBM, another non-conventional AM structure that features an isophthalic acid amide head group exhibited lower membrane binding for all concentrations compared to 1cM. In this case, the presence of the sterically bulky, diacid head group raises the CMC value and, thereby, inhibits formation of the membrane-binding micelles at the concentrations considered in this investigation.

The QCM-D measurements of the rate of AM mass deposition (Figure 5) revealed that the best overall binders also have high rates of interaction with the membrane. The resultant QSAR model reproduced the trend of membrane binding observed experimentally (Figure 6). The PLS linear regression produced a statistically significant correlation (*R*^2^ = 0.9, *R*^2^_cross-validated_ = 0.7) between the predicted and experimental membrane binding data. Decomposition of the loadings for the leading PLS terms into the original descriptors revealed that molecular size, polarizability, lipophilicity, and head-group charge figure prominently in explaining the relationship between AM structure and membrane binding affinity [31].

The QSAR model was tested externally using AMs 1cP and 1cAR, which were not included for model building. Corresponding values of the QSAR-predicted and experimental % (mass deposition) are proportionately self-consistent, which gives confidence in the predictive capacity of the model (Figure 7).

## 3. Experimental Section

### 3.1. Computational Modeling of AM Interactions with Model Membranes

#### 3.1.1. Overview of the Modeling Workflow

A set of 12 macromolecular structures was examined which vary with respect to their physicochemical features. This includes the nature of the terminal head group, hydrophobicity, carbohydrate structure, and stereochemistry. The AMs were constructed and energy minimized in Molecular Operating Environment (MOE), 2011.10 (Chemical Computing Group Inc., Montreal, Canada). Initial parameterization and charge distribution calculations for AM input structures for the MD simulations were performed with the *antechamber* program from the Amber Tools package [32]. Next, a two-tier process was implemented that comprised CG MD simulations on a three-dimensional model system to extract one or more representative (low energy) structures, followed by AA MD simulations to yield highly resolved AM conformers from which molecular descriptors were subsequently calculated.

#### 3.1.2. CG MD Simulation System

The multi-molecular ensemble for the study of the AM-lipid membrane interactions comprised a single AM molecule above an artificial lipid membrane comprised 512 dipalmitoylphosphatidylcholine (DPPC) molecules immersed in a saline bath containing approximately 20,000 CG waters together with Na^+^Cl^−^ at 120 mM. All components of the system were described using the CG MARTINI force field developed by Marrink *et al*. [9,33]. This accurately reproduces structural and thermodynamic properties of both lipid and macromolecule systems. In the MARTINI force field, groups of atoms are bundled into CG interaction sites, which are parameterized to retain the underlying molecular details of the corresponding atoms. Simulation beads are generally composed of four heavy atoms (excluding hydrogen) with an effective diameter of 0.47 nm. When a finer level of detail is necessary, as in the case of ring-like structures, 2–3 heavy atoms with a diameter of 0.43 nm represent an interaction site. Beads are grouped into four types: polar (P), nonpolar (N), apolar (C), and charged (Q) with further sub-types of each bead available to address the degree of polarity (1 = lower polarity to 5 = higher polarity) and the ability for hydrogen bonding (d = donor, a = acceptor, da = donor and acceptor, 0 = none). Non-bonded interactions between beads are treated with a shifted Lennard-Jones potential and electrostatic interactions are treated with a screened Coulomb potential [34]. Bond length and bond angle motions are described by harmonic potentials and dihedral constraints are implemented to prevent out-of-plane distortions of ring structures. The parameters for lipids, water, and ions are described by Marrink *et al.* [35]. The CG structure for each AM was created as follows: the parameters for the PEG portion of the molecule were taken from previous MARTINI studies on PEG [33]; the aliphatic arms on the AM backbone were parameterized as lipid tails. The AMs were parameterized as described previously [9,35]. Standard MARTINI parameters were used for the non-PEG portion of the molecules: bonded interactions (*R*_b_ = 0.47, *k*_b_ = 1250 kJ·mol^−1^·nm^−2^) and a harmonic angle potential was used to keep the aliphatic chains linear (θ = 180°, *k*_θ_ = 25 kJ·mol^−1^).

#### 3.1.3. CG MD Membrane-AM Interactions

The CG MD simulations were performed with the GROMACS software package version 4.5.5 [36]. In all simulations, in accordance with the MARTINI force-field, Lennard-Jones interactions were smoothly shifted to zero between a distance of 0.9 and 1.2 nm and electrostatics were smoothly shifted to zero between 0 and 1.2 nm. The non-bonded neighbor list was updated every 10 steps with a neighbor list cut-off of 1.4 nm. All simulations were performed in an isothermal-isobaric (NPT) ensemble with the system coupled to a Berendsen thermostat at 310 K and a coupling constant of τ_T_ = 1.0 ps [37]. Berendsen pressure coupling was used to maintain the system at a pressure of 1.0 bar using a coupling constant of τ_P_ = 2.0 ps and a compressibility of 3 × 10^−5^ bar^−1^. The integration time step was 10 fs. The CG nature of the MARTINI force field yields a smooth energy landscape for faster dynamics [9]. Simulations began with a single AM placed in solution above an equilibrated DPPC bilayer, as shown in Figure 2. Following a 20 ns equilibration period, the system was allowed to evolve in time for 1 μs. To obtain accurate statistics, 10 replicates were run for each AM. All analyses were performed using the GROMACS molecular dynamics software tools, and visualizations were created using Visual Molecular Dynamics (VMD) [38,39].

Starting with an AM placed in solution above the bilayer, simulations were run for all AMs to determine their propensity to spontaneously insert into the bilayer. The extent of insertion was quantified by measuring the distance from the AM head group to the bilayer center of mass (COM). The thickness of a DPPC bilayer in this system is approximately 4 nm. AM head group to bilayer COM distances < 2 nm occurring during a simulation were taken to be an insertion event. The next set of analyses characterized the interaction of AMs once they were inserted into membranes. AMs showing insertion events were analyzed to compute the orientation and distance of the AM PEG tail from the plane of the bilayer. The orientation was computed by defining a vector between the first PEG bead and the terminal PEG bead, then measuring the angle between this vector and the *z*-axis (perpendicular to the plane of the membrane). Distance distribution was measured by calculating the distance between the terminal PEG bead and the bilayer surface, defined by the average position of the DPPC phosphate groups or the average distance between all PEG beads and the bilayer surface.

#### 3.1.4. Calculation of the Potential of Mean Force

Umbrella sampling simulations were performed to calculate the free energy profiles (Potential of Mean Force) for 1cM, 0cM, and 1nM to understand the influence of head-group charge on AM interaction with a DPPC bilayer. In each case the AM was initially placed 7 nm above the bilayer, and a pulling simulation was carried out to bring the AM head group coincident with the bilayer center of mass at a rate of 0.0014 nm/ps. Seventy windows, spaced 0.1 nm apart, were extracted from this trajectory for use in umbrella sampling simulations. Each simulation window harmonically restrained the AM head-group at a fixed distance from the bilayer center of mass using a force constant of 1000 kJ·mol^−1^·nm^−2^. The simulation time per window was 50 ns with the first 20 ns discarded as equilibration. The Weighted Histogram Analysis Method implemented in the *g_wham* Gromacs analysis script was used to calculate the potential of mean force from the umbrella sampling simulations. All Potential of Mean Force plots were shifted to 0 kCal·mol^−1^ at a distance 5 nm from the bilayer surface.

#### 3.1.5. Reverse Mapping of CG Structures to All-Atom AM Structures

The reverse mapping technique of Rzepiela *et al.* was applied to convert the CG structures to AA structures [16,40]. The *Antechamber* module of the Amber Tools software package was used to generate atomistic topologies for each AM and the program *acpype* was used to convert Amber topologies into GROMACS format [32,41].

#### 3.1.6. All-Atom MD Simulations

Following CG MD simulation for 400 ns, the average low-energy structures of the AMs were further refined by AA MD simulations in aqueous solution over the surface of the membrane bilayer, constructed to mimic the macrophage cellular surface. Each reverse mapped AM was placed over the surface of the constructed membrane, and neutralized with sodium ions and solvated using the *tleap* subroutine in Amber 12 [32]. The AA MD simulations spanned 10 ns for each of the 12 AMs comprising 1 AM, 812 lipids and 82560 water molecules in a periodic box and were run on a dedicated high performance GPU Linux cluster [42].

#### 3.1.7. Molecular Descriptor Generation

Molecular descriptors associated with the physicochemical properties of the AMs were calculated for correlation with experimental membrane binding data. Descriptor generation was performed using MOE 2D topological and chemical group count descriptors. The process yielded 111 descriptors, including those pertaining to atomic and connectivity properties, atomic polarization, molecular weight and structure, molecular size, volume and surface area, as well as electrostatic and transport properties [43,44,45]. The Poisson-Boltzmann equation was used to calculate the electrostatic potential field and to visualize the charge distribution in MOE (Figure 8).

#### 3.1.8. QSAR Modeling

**Figure 9:**
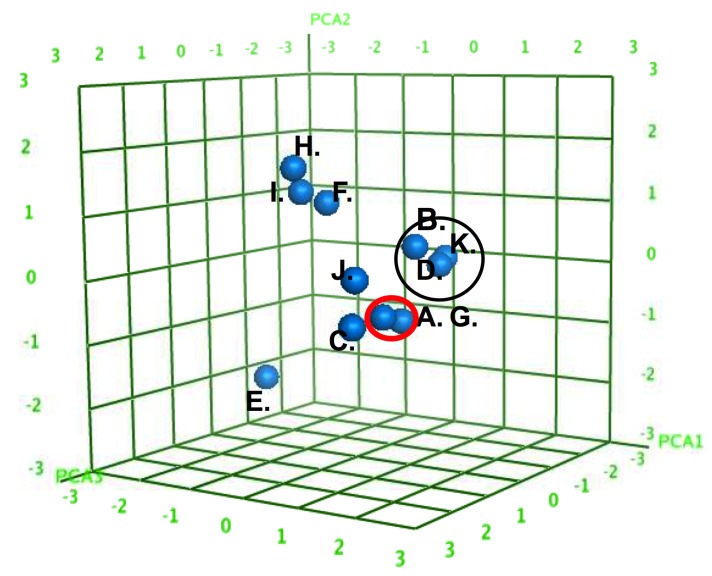
Three-dimensional scores plot of the AMs (A–J) in Principal Component space for PCA1, PCA2, and PCA3. The AMs divided into specific subclusters (denoted by circles) based on similar physicochemical features. Decomposition of the PCA loadings into the original descriptors found major contributions by the calculated log octanol/water partition coefficient (slogP); the total hydrophobic surface area (ASA_H); and the total polar surface area (ASA_P).

The QSAR model employs statistical regression methods to correlate changes in the values of specific calculated molecular descriptors of the AMs with changes in the corresponding experimentally measured binding affinity (Figure 9). Development of the eventual QSAR model ensues in two phases, known as training (model building) and testing (model validation). The training set consisted of ten AMs. Partial least squares (PLS) regression was employed to build a linear regression model. The initial model was tested internally using leave-1-out cross validation. The physicochemical features of the AMs were successfully discerned by physicochemical descriptor modeling. Figure 9 shows clusters of AMs possessing similar characteristics for structural and conformational comparison. Physicochemical descriptors are used to account for properties of the macromolecule serving as numerical descriptions or characterizations of structural features of the AMs including composition, spatial organization and surface area accessibility (Figure 9).

The QSAR model is a valuable tool for guiding new AM designs within the applicability domain of the training set and for predicting their membrane binding ability [46]. A preliminary QSAR model was constructed using PLS regression by correlating the membrane binding data retrieved from QCM-D with the subset of molecular descriptors. Predicting the membrane binding of two AMs, which were excluded from the training set, further validated the model. Analysis of the PLS regression equation identified those molecular descriptors that correlated most strongly with AM-membrane binding.

### 3.2. Experimental Studies of AM Binding to Model Membranes

#### 3.2.1. QCM-D Experimental Methods

The QCM-D measurements were performed on a Q-sense E4 instrument with quartz crystal sensors (Biolin Scientific, Sweden). The baseline was established for silicon dioxide coated quartz crystals equilibrated with 10 mM of HEPES buffer (MP Biomedical LLC, Aurora, OH, USA) at 37 °C at a flow rate of 0.92 mL min^−1^, The frequency (Δ*f*_n_) and dissipation changes were recorded over time for harmonics *n* = 3, 5, 7, 9 and 11. The change in mass (Δ*m*) was calculated using the Sauerbray equation Δ*m* = −CΔ*f*/*n* (*m*, mass deposition; Δ*f*, change in frequency of the quartz crystal; *C*= 17.7 ng·cm^−2^ Hz^−1^ is the quartz crystal constant) [47]. To evaluate the AM-membrane bilayer interaction, viscoelastic modeling was performed using the Voigt model for viscoelastic materials that consists of a spring and dashpot in parallel. The mass and dissipation sensitivities of QCM-D measurements in liquid were approximately 1.8 ng/cm^2^ and 0.1 × 10^−6^ D (dissipation parameter), respectively, according to the manufacturer of the instrument, Biolin Scientific. The rate of mass deposition was obtained by linear regression after equilibration.

QCM sensors: Silicon dioxide QSX 303 SiO_2_ sensor crystal was obtained from Q-sense Incorporated with fundamental frequency of 5 Hz (QSX 303, LOT Oriel Group, Germany). Pico purified water was used to prepare buffers, HEPES, MP Biomedical LLC (Aurora, OH, USA)

#### 3.2.2. Liposomes

Lipids: (1,2-dioleoyl-3-trimethylammonium-propane (DOTAP) and 1,2-dioleoyl-sn-glycero-3-phosphatidylethanolamine (DOPE) lipids were obtained from Avanti Polar Lipids (Alabaster, AL, USA). Lipids were dissolved in chloroform and dried at room temperature using a rotary evaporator, followed by drying overnight in vacuum at room temperature. Lipid film was hydrated using 10 mM HEPES, MP Biomedical LLC (Aurora, OH, USA) for 1 mg/mL solutions with continuous shaking on an orbital reciprocating shaker for 1 h. Liposomes were produced by extruding through an Avanti mini-extruder system (Avanti Polar lipids) using 0.1 µm membrane filters, resulting in sizes averaging 100 nm after 21 passes. Liposomes were used within 10 h, stored at 4 °C [48,49]. Liposome sizes were measured using a Dynamic Light Scattering (DLS), NanoZS90 Instrument (Malvern Instruments, Malvern, UK). Three 1 mL samples were evaluated, with 15 runs in triplicate. Values reported represent the average of the three samples. For the QCM-D experiments, the model lipid bed was constructed with DOPE-DOTAP lipids due to the necessity of a stable lipid bilayer construction on the quartz crystal [48]. DOPE is zwitterionic and DOTAP possesses a positive charge.

#### 3.2.3. Amphiphilic Macromolecules

Macromolecule synthesis and structural characterization were carried out as described previously [6,10,50]. Materials used for synthesis include 5 kD heterobifunctional tBOC-protected PEG, Layson Bio Inc. (Arab, AL, USA). All other reagents and solvents were purchased from Sigma-Aldrich (St. Louis, MO, USA) and used without further purification. Chemical structure and compositions were confirmed by ^1^H and ^13^C NMR spectroscopy with CDCl_3_-d solvent on a Varian 400 MHz spectrometer. Macromolecule molecular weight and polydispersity index were determined using gel permeation chromatography. Zeta potential and NAP size was measured using the Malvern Instrument, Nano ZS-9 Zetasizer. Lyophilized AM stock hydrated using 10mM HEPES MP Biomedical LLC (Aurora, OH, USA) to concentrations of 1 × 10^−5^ M, 1 × 10^−6^ M, 1 × 10^−7^ M and 1 × 10^−8^ M.

#### 3.2.4. Bilayer Formation and AM Exposure

HEPES buffer was flowed across QCM-D crystals at 0.92 mL/min for approximately 5 min until the frequency and dissipation were stable for baseline. The liposome solution was then allowed to flow across the crystal surface at flow rate of 5 mL/min for ~15 min to form a Supported Lipid Bilayer (SLB) [48]. This was confirmed via mass deposition QCM-D measurements. The rates were optimized based on several trials for bilayer consistency. The sensor crystals were washed with HEPES buffer (10 mL) to remove unbound lipids. Once a stable baseline was accomplished, AM solution was added for up to 1 h under gentle flow of 0.92 mL/min. The AM solution was then replaced by a final buffer rinse at 0.92 mL/min for 5 min.

#### 3.2.5. Statistical Analysis

Statistical analyses were carried out using a one-way ANOVA test. The significance criteria assumed a 95% confidence level (*P* < 0.05). Standard error of the mean is reported in the form of an error bar of the final data.

## 4. Conclusions

Through the combined application of modeling and experimental techniques, we elucidated the minute variations in the behaviors of the various carbohydrate-derived amphiphilic macromolecules (AM) at the interface of lipid membranes and membrane-mimetics. We identified and defined the effects of specific structural design elements of the AM library, such as stereochemistry, charge and amphiphilicity. This structure-activity relationship is particularly evident in the analysis of experimental membrane binding. The combined efforts of both simulation and experimentally modeled membrane binding complemented the rational macromolecule design. MD simulations provided an opportunity to observe the binding behavior of a macromolecule in a bio-relevant environment. The MD and QCM-D studies have led to structural activity determination in the context of membrane binding. Insertion of the carbohydrate-based hydrophobic segment into the fluid phase bilayers, potentially anchors the AM to the surface of the membrane, which serves as a primary mechanism of membrane association. Liposomal binding was demonstrable in previous assays; however, previous to the present analysis there were no direct methods for measurement of physicochemical properties in the setting of the ubiquitous membrane. The simulations highlight molecular details of membrane interfacial interactions and provide design principles for structure activity.

The QSAR model established a pathway for the rational design of future macromolecules. The four most explanatory molecular descriptors were of the two-dimensional (2D) category, suggesting that relatively simple chemistry-based properties are sufficient to capture the properties of membrane binding of the AMs. We established that coordinated use of coarse-grained (CG) and all-atom (AA) MD simulations on multicomponent ensembles of AM model systems offers an efficient and effective computational procedure for simulating AM-membrane interactions and binding that complements interpretation of the QCM-D experiments. A QSAR model was constructed by correlating variations in the experimentally determined membrane binding (%mass deposition) of this series of AMs. Changes in their molecular structures and physicochemical features as represented by calculated molecular descriptors were determined using PLS linear regression. Decomposition of the PLS regression loadings indicates that membrane binding is affected by specific physicochemical properties of the AMs, notably the stereochemical arrangement of their aliphatic arms, the electrostatic charge of their terminal head group, and their hydrophilic-lipophilic balance. The QSAR model, together with knowledge of these structure-based features, will help to guide the design of next-generation AMs with tailored membrane binding capacity for therapeutic applications.

One of the most remarkable variations detected was between nearly identical AMs with one aliphatic arm stereochemistry variant, 1cM *versus* 1cS. When considering the molecular model of 1cS, the aliphatic chains orientation provides less access to the charge compared to the exposed charge observed for 1cM. The highest rate of deposition was observed for 1cM and L-1cT. Neutral molecules were observed to have reduced binding relative to comparative AMs. Thus, the various approaches presented here offer insights to compare and characterize the biocompatibility of AMs with prescribed properties. These approaches additionally provide tools for an in depth study of AMs, which can guide rational development of functionally tailored AM biomaterials. These considerations may also influence the strategy for synthesis and functional modifications of AMs as these could exert a major effect on the activity of the bioactive. Insights from this study are relevant to screening AMs for specific applications, for example, for solubilizing bioactive pharmacologic factors [51], or leveraging the inherent bioactivity of the macromolecules for regulating lipoprotein trafficking into inflammatory cells [16].

In summary, the present study establishes the combined impacts of MD simulations and membrane binding experiments in rational macromolecule design. The simulations, and the subsequent QSAR model, provide a molecular-level understanding of the AMs physicochemical properties. Thus, mapping the structural influence on binding to membrane lipid bilayers is a robust approach to define complex macromolecular structures. Future studies of the model system presented in this work could serve as a platform for the rational design and screening of next generation AMs with improved performance qualities. Furthermore, the QSAR models will gain in predictive performance and statistical robustness as AM-based biomaterials are transplanted and evaluated as therapeutic candidates *in vivo* [52].
